# Antioxidant and hepatoprotective effects of *Artemisia dracunculus* against CCl_4_-induced hepatotoxicity in rats

**Published:** 2018

**Authors:** Vahid Zarezade, Jalal Moludi, Mostafa Mostafazadeh, Mohammad Mohammadi, Ali Veisi

**Affiliations:** 1 *Behbahan Faculty of Medical Sciences, Behbahan, Iran*; 2 *Nutrition Research Center, Department of Biochemistry and Diet Therapy, Faculty of Nutrition, Tabriz University of Medical Sciences, Tabriz, Iran*; 3 *Students’ Research Committee, Tabriz University of Medical Sciences, Tabriz, Iran*; 4 *Department of Biochemistry and clinical laboratories, Faculty of Medicine, Tabriz University of medical sciences, Tabriz, Iran*; 5 *Department of Biology, Faculty of Science, Shahid Chamran University, Ahvaz, Iran*

**Keywords:** Antioxidant, Artemisia dracunculus, Carbon Tetrachloride, Hepatotoxicity, Oxidative stress, Rats

## Abstract

**Objective:**

The present study was conducted to investigate the antioxidant and hepatoprotective activity of the hydro-alcoholic extract of aerial parts of *Artemisia* *dracunculus *(HAAD) against CCl_4_-induced hepatotoxicity in rats.

**Materials and Methods:**

The antioxidant activity was evaluated by reducing power, 2, 2-diphenyl-1-picrylhydrazyl (DPPH) and 2, 20-azino-bis (3-ethylbenzothiazoline-6-sulfonic acid) (ABTS) assays. Rats were pre-treated with either 50, 100, and 200 mg/kg of HAAD or silymarin (100 mg/kg; served as the positive control group) for 15 days and they received a single dose of CCl_4_ on the last day. Hepatoprotective effects were investigated by assessment of serum biochemical enzymes such as alanine transaminase (ALT), aspartate transaminase (AST), alkaline phosphatase (ALP), total protein (TP), total bilirubin (TB), malondialdehyde (MDA), and antioxidant enzymes (SOD, CAT, GST and GSH), along with histopathological studies.

**Results:**

Total phenolic content was 197.22±3.73 mg gallic acid equivalent/g HAAD dry weight. HAAD indicated powerful activity in FRAP, DPPH and ABTS tests. Acute toxicity study showed that the extract had an LD_50_ of >5000 mg/kg. Oral treatment with HAAD exhibited a significant decrease in the levels of AST, ALT, ALP and TB and an increase in the level of TP. The extract significantly diminished MDA levels. The activities of the antioxidant enzymes were significantly augmented in rats pretreated with HAAD 200 mg/kg. Histopathological examination demonstrated lower liver damage in HAAD-treated groups as compared to CCl_4_ groups.

**Conclusion:**

Our findings indicated hepatoprotective effects of the hydro-alcoholic extract of *A. dracunculus* on CCl_4_-induced hepatic damage in rats and suggested that these effects may be produced by reducing oxidative stress.

## Introduction

Carbon tetrachloride (CCl_4_) as a standout amongst the hepatotoxins, is generally utilized to induce experimental models (Cheng et al., 2013 ▶). It is accepted that CCl_4_ hepatotoxicity occurs due to its reductive dehalogenation reaction which is catalyzed by cytochrome P-450 in hepatocytes (Raj and Gothandam, 2014 ▶). This response actuates the generation of a few sorts of reactive oxygen species (ROS) (Jalali Ghassam et al., 2014 ▶). These ROS can bind to lipids or proteins resulting in creation of distinctive radicals and initiation of lipid peroxidation which end up in membrane injury and consequently damage the liver (Ali et al., 2014 ▶; Kepekçi et al., 2013 ▶). 

Today, administration of conventional or synthetic drugs for the treatment of liver disorders is facing serious side effects (Toori et al., 2015 ▶, Manns et al., 2006 ▶). Therefore it is necessary to seek for new medicines for liver disease, notably those originated from natural resources. Medicinal plants are great sources of natural compounds such as phenolic acids and flavonoids, which have been shown good *in vitro* antioxidative properties and are able to scavenge free radicals and defend liver from CCl_4_-induced hepatic injury (Azeem et al., 2010 ▶, Gnanadesigan et al., 2011 ▶, Gupta et al., 2011 ▶, Singhal and Gupta, 2012 ▶). 

*Artemisia dracunculus* L. (tarragon) is a perennial plant belonging to the Asteraceae family, which has a long history of use as a spice and remedy (Obolskiy et al., 2011 ▶). *A. dracunculus* is distributed over most of temperate Asia, western North America and central Europe (Eisenman and Struwe, 2011 ▶). Aerial parts of tarragon which have been used as an antihyperlipidemic and anticoagulant agent, are commonly consumed in fresh or dried form in salads and soups in Iranian cuisine Shahriyary and Yazdanparast, 2007 ▶). Previous studies on *A. dracunculus* indicated that this plant have bioactive constituents like α-pinene, terpinolene, allo-ocimene, α-trans-ocimene, 7-methoxycoumarin, trans-anethole, methyleugenol, estragole, γ-terpinene and elemicin (Aglarova, 2006 ▶, Khodakov et al., 2009 ▶, Kordali et al., 2005 ▶, Pappas and Sturtz, 2001 ▶, Sayyah et al., 2004 ▶). Moreover, several biological activities of tarragon such as antibacterial, antiplatelet, anti-inflammatory, antihyperglycemic, gastroprotective and antioxidant properties have been confirmed in the literature (Aglarova, 2006 ▶, Benli et al., 2007 ▶, Kheterpal et al., 2010 ▶, Lopes-Lutz et al., 2008 ▶, Shahriyary and Yazdanparast, 2007 ▶). 

To our knowledge, there is no report on the antioxidant and hepatoprotective potential of* A. dracunculus* in the literature. Hence, the aim of this study was to analyze the antioxidant and hepatoprotective activity of hydro-alcoholic extract of aerial parts of *A. dracunculus* (HAAD) against CCl_4_-induced hepatotoxicity in rats. 

## Materials and Methods

This experimental study was carried out in Yasuj University of Medical Sciences, Yasuj, Iran from December 2014 to March 2015. The study was approved by the Ethics Committee of Yasuj University of Medical Sciences (Code: 94/6/2/5) in August 2014 and was exactly performed according to the “Principles of Laboratory Animal Care” (NIH Publication No. 86-23).

**Chemicals and reagents**

Trichloroacetic acid (TCA), Thiobarbituric acid (TBA), pyrogallol, Folin–Ciocalteu reagent, gallic acid, carbon tetrachloride (CCl_4_), potassium persulfate, potassium ferricyanide, and diethyl ether were obtained from Merck, Germany. Silymarin, 2, 2-diphenyl-1-picrylhydrazyl, 2, 20-azino-bis (3-ethylbenzothiazoline-6-sulfonic acid), 5, 5'-dithiobis-(2-nitrobenzoic acid, and ascorbic acid were purchased from Sigma-Aldrich (St. Louis, MO, USA). The kits for the determination of ALT, AST, ALP, TB and TP were purchased from Pars Azmun, Tehran, Iran.

All chemicals and reagents used for biochemical assays were of analytical grade. 

**Plant materials**

Aerial parts of *A. dracunculus*, including stems and leaves were collected in August 2014 from the suburbs of Behbahan, Khuzestan province, Iran. The plant was authenticated by Dr. J. Vaezi from Department of Biology, Faculty of Sciences, Shahid Chamran University of Ahvaz, Ahvaz, Iran, and a voucher specimen (herbarium No. 93/135) was deposited. The collected aerial parts of *A. dracunculus* were shade dried, and pulverized.

**Extract preparation**

The powdered plant material (200 g) was concentrated for two times using l000 ml mixture of EtOH-H_2_O (7:3) at 45°C for 48 hr. The extract was separated and organic solvent was totally dissipated under decreased pressure in a rotary evaporator (Hyedolph, type: Heizbad Hei-VAP, Germany) at 60°C. Then, the concentrated extract was dried at room temperature. The average yield of the hydro-alcoholic extract was around 20.3% (Sadeghi et al., 2014 ▶).

**Determination of total phenolic contents**

The Folin–Ciocalteu method was applied for determination of total phenolic contents in HAAD (Roby et al., 2013 ▶). First, 50 µl of the extract (200 μg/ml) was blended with 5 ml Folin–Ciocalteu reagent and then, 10 ml of saturated soda (Na_2_CO_3_) was added to this mixture. The tubes were vortexed or 15 sec and incubated in dark for about 30 min for color emersion. The absorbance of the tubes was then measured at 760 nm using a Cary 5000 UV-Vis-NIR spectrophotometer. Also, in order to prepare the standard curve, gallic acid at different concentrations was used and the absorbance was measured at 760 nm. Trials were carried out in triplicate and the average was reported. The total phenolic amount (presented as mg gallic acid/g HAAD) of the *A. dracunculus* was obtained from the standard gallic acid curve and following equation:

Absorbance = 0.0008 × gallic acid (mg)

**FRAP (ferric reducing antioxidant power) assay**

The reducing power of HAAD was determined by the method of Ilhami Gulcin et al. (Gülçin et al., 2010 ▶) with slight modifications. Different concentrations of ascorbic acid (as positive control) and HAAD (100-500 µg/mL) in 1 mL of distilled water were blended with phosphate-buffered saline (PBS) (2.5 mL, 0.2 M, pH 6.6) and potassium ferricyanide (K_3_Fe(CN)_6_) (2.5 mL, 30 mM). Then, the mixture was incubated at 50 ^o^C for 20 min. Afterwards, 2.5 mL trichoroacetic acid (TCA) (0.6 M) was added to the solution and centrifuged at 3000 g for 10 min. After the addition of distilled water (2.5 ml) and 0.5 ml FeCl_3_ (6 mM) to the upper layer of the mixture, the absorbance of the solution was assayed at 700 nm using a spectrophotometer. All assays were repeated three times. The absorbance of the reaction mixture has a direct correlation with the reduction capacity.

**DPPH free radical scavenging activity**

DPPH^•^ free radical scavenging ability of the plant extract was determined according to the method described by Dehong Hua et al. (Hua et al., 2014 ▶) with minor modifications. For this purpose, 1 ml of different concentrations of the extract (25, 50, 100, 200 and 400 µg/ml) were added to 1 ml of a 0.1 mM DPPH methanolic solution and the mixture was incubated for 30 min in dark. Next, the absorbance of the solutions was measured at 515 nm. Ascorbic acid was utilized as positive control and the same procedure was carried out. All assays were performed in triplicate. The scavenging activity was calculated using the following equation (Zhang et al., 2015 ▶):

DPPH radical scavenging activity (%) = $[1-\frac{\mathrm{As}-\mathrm{Ab}}{\mathrm{Ac}}]\times 100$

Where, As: the absorbance of the test sample (DPPH solution + extract and ascorbic acid), Ab: the absorbance of the blank and Ac: the absorbance of the control (DPPH solution without samples).

**ABTS radical scavenging activity**

ABTS radical cation scavenging activity of the extract was measured as antioxidant potential (Debbache et al., 2014 ▶). First, ABTS (7 mM) and potassium persulfate (2.45 mM) solutions were mixed and stirred at room temperature for 16 hr. Next, the stock solution was diluted with ethanol to attain an absorbance of 0.7 ± 0.02 at 750 nm. Thereafter, 100 μl of ABTS^+•^ solution was added to 100 μl of different concentrations (100-500 μg/ml) of HAAD and ascorbic acid (as positive control). Afterwards, the samples were vortexed and incubated in dark for 6 min. All assays were done in triplicate. ABTS radical-scavenging activity was evaluated by spectrophotometer at 750 nm and reported using the following equation: 

ABTS radical-scavenging activity (%) = $(1-\frac{\mathrm{As}}{\mathrm{Ac}})\times 100$

Where, As: the absorbance of the test sample (ABTS solution + extract and ascorbic acid) and Ac: the absorbance of the control (ABTS solution without samples).

**Animals and treatment**

Adult male Wistar rats weighing 200-250 g were utilized in this study. The experimental procedures were exactly performed according to the “Principles of Laboratory Animal Care” (NIH Publication No. 86-23). The animals were randomly divided into groups of six rats. The rats were kept under 12 hr:12 hr light/dark cycles at 24 ± 2°C and permitted to have free access to normal diet and water. The animals were adapted to laboratory conditions for 7 days prior to initiation of examination.

**Acute toxicity test**

In order to measure the LD_50_ of the HAAD, three doses of the extract (1000, 3000, and 5000 mg/kg) were given orally to three groups of the rats (n=6). The animals were supervised for behavioral changes and mortality for 48 hr (Kumar et al., 2012 ▶).

**CCl**_4_ **-induced hepatotoxicity**

In this study, we used the method of induction of hepatotoxicity using CCl_4_which was previously described by Raj et al. (Raj and Gothandam, 2014 ▶). The animals were randomly allocated into six groups (six rats per each group) including group I (normal control group), group II (CCl_4_ group) (both groups I and II received normal saline p.o. every day), group III (positive control group) received silymarin (100 mg/kg per day p.o.) as the standard drug, groups IV, V and VI orally received HAAD 50, 100 and 200 mg/kg BW, respectively. Fifteen days after the onset of HAAD and silymarin administration, liver injury was induced by CCl_4_ (10 ml/kg of 1:1 v/v mixture of CCl_4_ and olive oil; i.p.) one hour after the last administration in all groups except for the normal control group. Then, 24 hr post CCl_4_ administration, all animals were euthanized by diethyl ether and blood samples were collected promptly from the abdominal aorta. The liver was dissected and excised immediately. A section of the left lobe of the liver was used for histopathological studies, and the remaining was frozen quickly and stored at -80 ^o^C for biochemical analysis. 

**Assessment of liver function parameters**

Collected blood samples were centrifuged at 2500 rpm at room temperature for 20 min and serum was separated. The serum levels of biochemical parameters namely, ALT, AST, ALP, TP and TB were evaluated using commercially available diagnostic kits (Roche Diagnostics) and Cobas C 311 autoanalyzer (Roche Hitachi, Manheim, Germany) (Zeashan et al., 2008 ▶).

**Assessment of lipid peroxidation**

Thiobarbituric acid reactive substance (TBARS) assay was used for evaluation of MDA, an end-product of lipid peroxidation, as previously reported with minor changes (Pareek et al., 2013 ▶). The frozen portions of liver were thawed and homogenized in ice-cold phosphate buffer 50 mM (1:9, pH 7.4). Thereafter, the homogenate was centrifuged at 12000 rpm at 4 ^o^C for 15 min and the supernatant was collected in Eppendorf microtubes. Next, 1 mL of ice-cold TCA (75 mg/mL) was mixed with 1 mL of the homogenate and then centrifuged at 1500 rpm. Afterwards, 1 mL of TBA (8 mg/mL) was added to the supernatant and the mixture was kept in boiling water for 45 min. The absorbance of the samples was measured at 535 nm. The results were expressed as nmol MDA per g tissue.

**Assay of glutathione (GSH)**

The reduced glutathione content of liver tissue was determined by the method of Raj et al. (Raj and Gothandam, 2014 ▶) with slight modifications. In order to precipitate the proteins, 1 ml of liver homogenate was mixed with 1 ml of 10% TCA and incubated for 5 min. Then, the mixture was centrifuged at 2000 rpm at 4°C for 10 min. Afterwards, 0.5 ml of supernatant was mixed with 5 ml of 0.2 M PBS (pH 8) and 2 ml 0.6 mM Ellman's reagent (5, 5'-dithiobis-(2-nitrobenzoic acid)) or DTNB at 4°C for 5 min. The absorbance was measured at 412 nm, and the concentration of GSH was expressed as μg/mg protein.

**Assessment of superoxide dismutase (SOD), catalase (CAT) and glutathione-S-transferase (GST) activities**

SOD activity was measured using autoxidation of 2mM pyrogallol in Tris buffer (Raj and Gothandam, 2014 ▶). Results are shown as unit/min/mg protein. One unit (U) of SOD activity was defined as the amount of enzyme required to inhibit the oxidation of 50% pyrogallol. Disintegration of H_2_O_2_ in the presence of CAT was recorded at 620 nm (Maheshwari et al., 2011 ▶). Results are expressed as µmole H_2_O_2_/min/mg protein. The activity of GST was assessed in PBS (0.1 M, pH 7.6) containing glutathione (0.5 mM) and 1-chloro-2, 4-dinitrobenzene (CDNB) 0.5 mM) (Jalali Ghassam et al., 2014 ▶). The change in absorbance at 344 nm was monitored using a spectrophotometer. Results are presented as mmole CDNB conjugate/min/mg protein.

**Histopathological studies**

For histological assessment, the liver samples from all groups were taken and fixed in 10% formaldehyde for 1 week. Thereafter, fixed tissues were embedded in paraffin and cut into 3-4 μm thick sections. The sections were stained using hematoxylin and eosin and observed by a blind pathologist. 

**Statistical analysis**

The results are expressed as mean ± standard error of mean (Mean ± SEM). The data were analyzed by ANOVA followed by Tukey as *post-hoc* test, using SPSS 22.0 software. A p< 0.05 was assumed significant.

## Results

**Total phenolic content**

Total phenolic content was 197.223±3.73 mg gallic acid equivalent/g HAAD dry weight. The result was the Mean±SEM of three times of absorbance reading.

**Ferric reducing antioxidant power activity**

**Figure 1:**
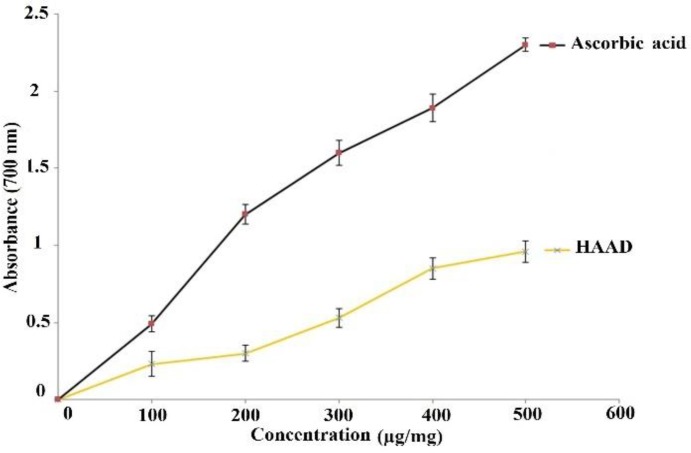
*In vitro* antioxidant activity of HAAD and ascorbic acid. Graph showing ferric reducing antioxidant power activity. Values are presented as mean ± SEM. HAAD: hydro-alcoholic extract of aerial parts of *A. dracunculus*

As shown in Figure 1, different concentrations of HAAD (100-500 µg/ml) exhibited powerful reducing power in a dose-dependent manner. Also, ascorbic acid at the same concentrations showed effective reducing power more than that of HAAD.

**DPPH radical scavenging activity**

**Figure 2:**
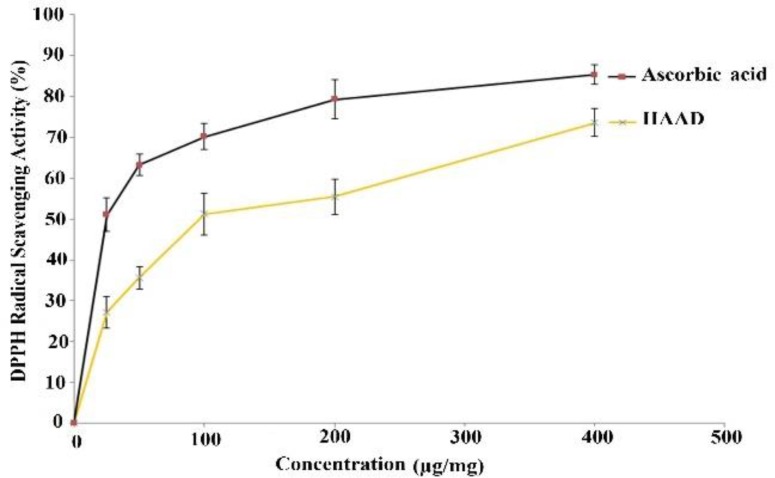
. *In vitro* antioxidant activity of HAAD and ascorbic acid. Graph showing DPPH radical scavenging activity. Values are presented as mean ± SEM. HAAD: hydro-alcoholic extract from aerial parts of *A. dracunculus*

The DPPH radical scavenging activity of the plant is expressed as percentage. As illustrated in Figure 2, disparate concentrations of HAAD (25, 50, 100, 200 and 400 µg/ml) showed marked DPPH radical scavenging activity almost comparable with that of ascorbic acid at the same concentrations of those of samples.

**ABTS radical scavenging activity**

**Figure 3:**
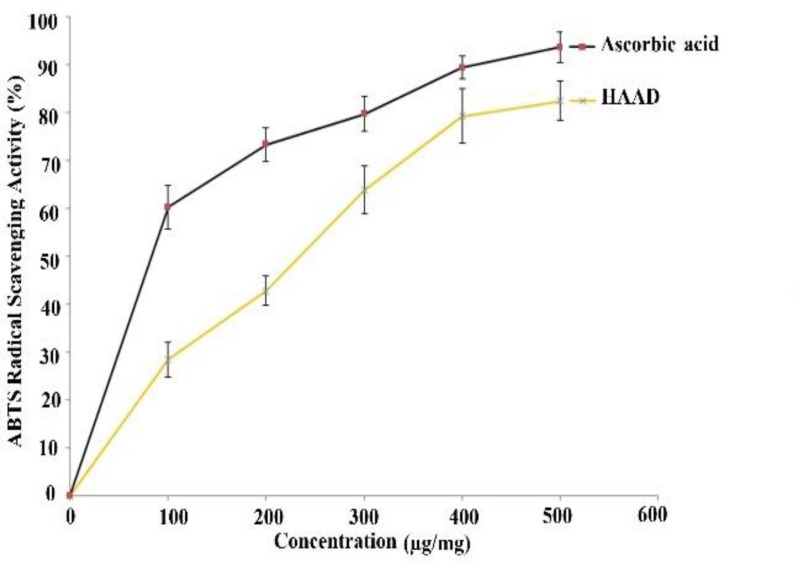
*In vitro* antioxidant activity of HAAD and ascorbic acid. Graph showing ABTS radical scavenging activity. Values are presented as mean ± SEM. HAAD: hydro-alcoholic extract of aerial parts of *A. dracunculus*

The results of ABTS radical scavenging activity of different concentrations of the extract (100-500 µg/ml) are presented in Figure 3. HAAD exhibited powerful scavenging activity but it was lower than that of ascorbic acid at the same concentrations.

**Acute toxicity**

The HAAD did not cause any behavioral changes or mortality during 48 hr. These results showed that the LD_50_ of the HAAD was >5000 mg/kg.

**Effect of HAAD on serum biochemical enzymes**

**Figure 4:**
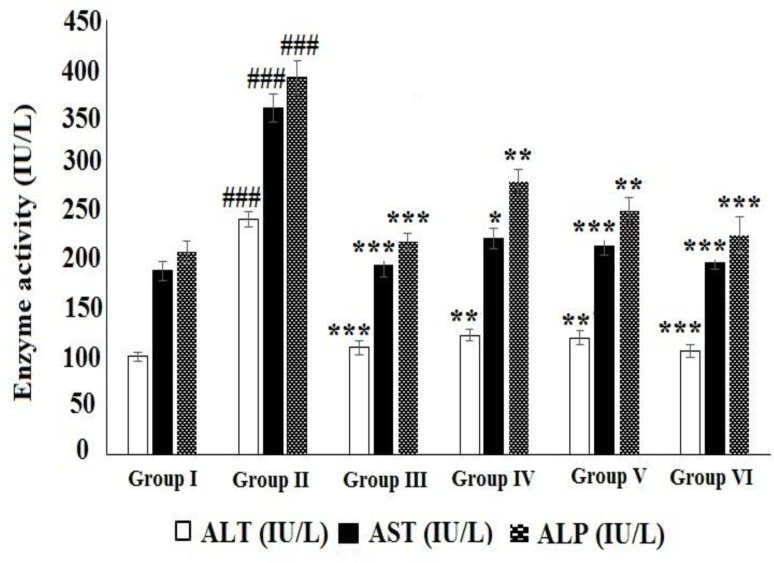
Effect of HAAD and silymarin on ALT, AST, and ALP activity in CCl_4_-induced hepatotoxicity. Groups – I: normal saline; II: CCl_4_ (CCl_4_ group); III: Silymarin (100 mg/kg) + CCl_4_ (10 ml/kg); IV: HAAD (50 mg/kg) + CCl_4_ (10 ml/kg); V: HAAD (100 mg/kg) + CCl_4_ (10 ml/kg); VI: HAAD (200 mg/kg) + CCl_4_ (10 ml/kg). Values are mean ± S.E.M. (n=6). ^###^p<0.001 vs. group (I); ^*^p<0.05, ^**^p<0.01, and ^***^p<0.001 vs. CCl_4_ group (II).

**Figure 5:**
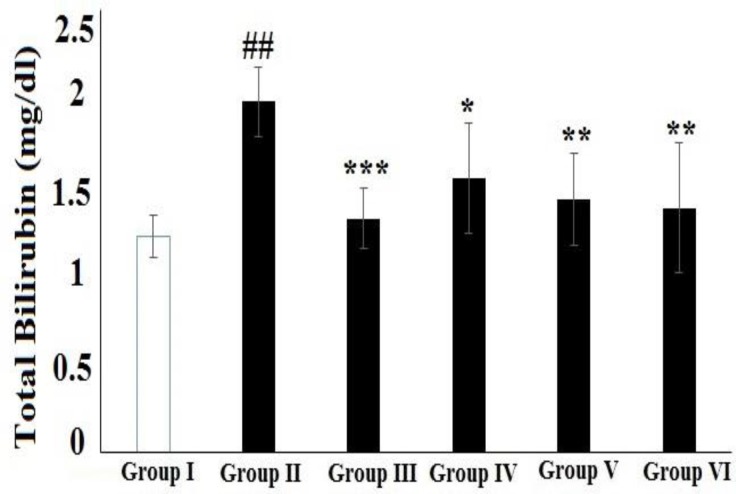
Effect of HAAD and silymarin on total bilirubin (TB) in CCl_4_-induced hepatotoxicity. Groups – I: normal saline; II: CCl_4_ (CCl_4_ group); III: Silymarin (100 mg/kg) + CCl_4_ (10 ml/kg); IV: HAAD (50 mg/kg) + CCl_4_ (10 ml/kg); V: HAAD (100 mg/kg) + CCl_4_ (10 ml/kg); VI: HAAD (200 mg/kg) + CCl_4_ (10 ml/kg). Values are mean ± S.E.M. (n=6). ^##^p<0.01 vs. group (I); ^*^p<0.05, ^**^p<0.01, and ^***^p<0.001 vs. CCl_4_ group (II

**Figure 6:**
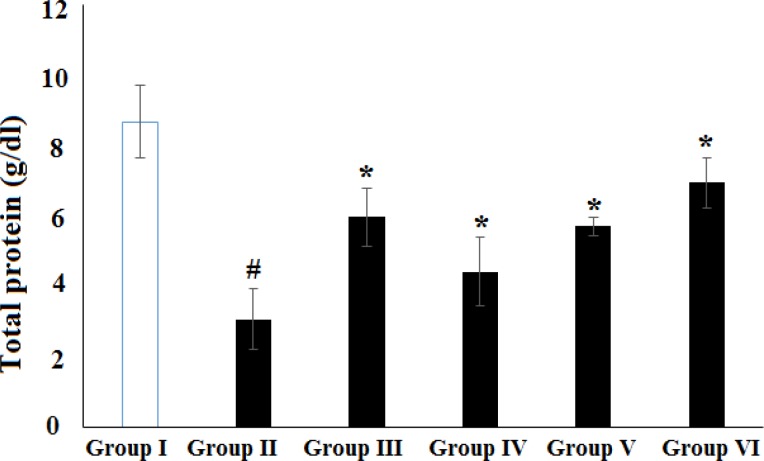
Effect of HAAD and silymarin on total protein (TP) in CCl_4_-induced hepatotoxicity. Groups – I: normal saline; II: CCl4 (CCl_4_ group); III: Silymarin (100 mg/kg) + CCl_4_ (10 ml/kg); IV: HAAD (50 mg/kg) + CCl_4_ (10 ml/kg); V: HEAD (100 mg/kg) + CCl_4_ (10 ml/kg); VI: HAAD (200 mg/kg) + CCl_4_ (10 ml/kg). Values are mean ± S.E.M. (n=6). ^#^p<0.05 vs. group (I); ^*^p<0.05 vs. CCl_4_ group (II

The effects of HAAD on serum biochemical parameters of CCl_4_-poisoned rats are shown in Figures 4, 5, and 6. The levels of AST, ALT, ALP and TB significantly increased while TP level significantly decreased in CCl_4_ group compared to normal group. Oral treatment of the rats with HAAD 50, 100 and 200 mg/kg showed a significant decrease in the levels of serum enzymes namely, AST, ALT, ALP and TB (p<0.05) while induced a significant increase in the level of serum TP (p<0.05) compared to the CCl_4_ group. Oral administration of silymarin (100 mg/kg) also showed a significant decrease in the levels of serum ALT, AST, ALP and TB (p<0.001) while significantly increased the level of serum TP (p<0.05) compared to the CCl_4_ group.

**Effect of HAAD on liver lipid peroxidation and antioxidant enzymes**

**Figure 7.**
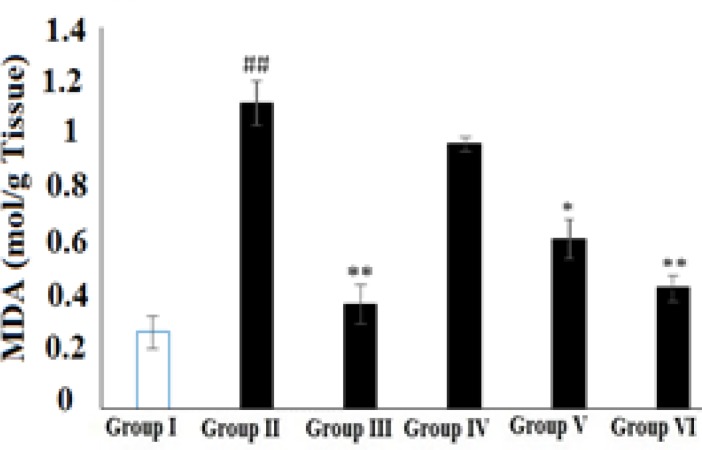
Effect of HAAD and silymarin on total MDA levels in CCl_4_-induced hepatotoxicity. Groups – I: normal saline; II: CCl_4_ (CCl_4_ group); III: Silymarin (100 mg/kg) + CCl_4_ (10 ml/kg); IV: HEAD (50 mg/kg) + CCl_4_ (10 ml/kg); V: HAAD (100 mg/kg) + CCl_4_ (10 ml/kg); VI: HAAD (200 mg/kg) + CCl_4_ (10 ml/kg). Values are mean ± S.E.M. (n=6). ^##^p<0.01 vs. group (I); ^**^p<0.01 and ^*^p<0.05 vs. CCl_4_ group (II

As illustrated in Figure 7, the level of tissue MDA significantly increased in the CCl_4_ group as compared to the normal group (p<0.01). The elevated level of tissue MDA was diminished significantly by the pretreatment of rats with HAAD at doses of 100 and 200 mg/kg as compared to the CCl_4_ group (p<0.05 and p<0.01, respectively). Silymarin 100 mg/kg also produced a significant reduction in MDA level compared to the CCl_4_ group (p<0.01). 

**Figure 8:**
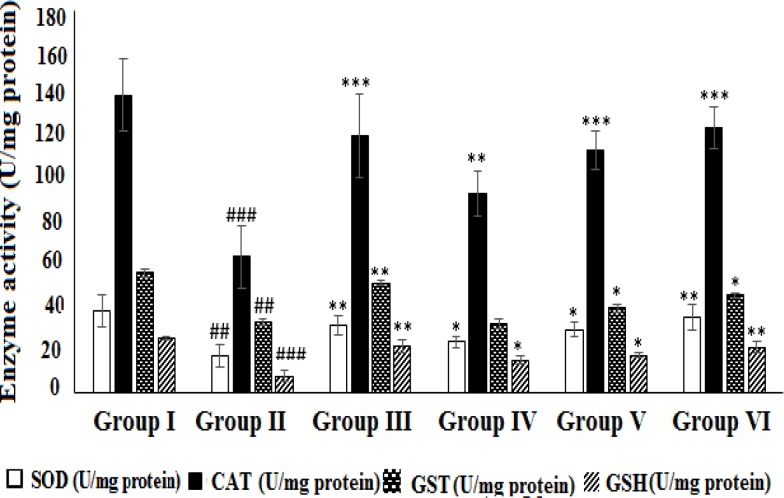
Effect of HAAD and silymarin on SOD, CAT, GST, and GSH activity in CCl_4_-induced hepatotoxicity. Groups – I: normal saline; II: CCl_4_ (CCl_4_ group); III: Silymarin (100 mg/kg) + CCl_4_ (10 ml/kg); IV: HAAD (50 mg/kg) + CCl_4_ (10 ml/kg); V: HAAD (100 mg/kg) + CCl_4_ (10 ml/kg); VI: HAAD (200 mg/kg) + CCl_4_ (10 ml/kg). Values are mean ± S.E.M. (n=6).^ ##^p<0.01 and ^###^p<0.001 vs. group (I); ^*^p<0.05, ^**^p<0.01, and ^***^p<0.001 vs. CCl_4_ group (II

The activities of the hepatic antioxidant enzymes (SOD, CAT and GST) and the level of glutathione were found to be drastically decreased (p<0.01 and p<0.001) in CCl_4_ group as compared to normal group (Figure 8). The decreased activities of the enzymes in CCl_4_-intoxicated rats were augmented more effectively (p<0.05, p<0.01 and p<0.01) in rats pretreated with HAAD 200 mg/kg , as compared to those pretreated with HEAD 50 and 100 mg/kg. This protective effect was more marked (p<0.01 and p<0.001) in animals pretreated with silymarin 100 mg/kg.

**Histopathological studies**

**Figure 9:**
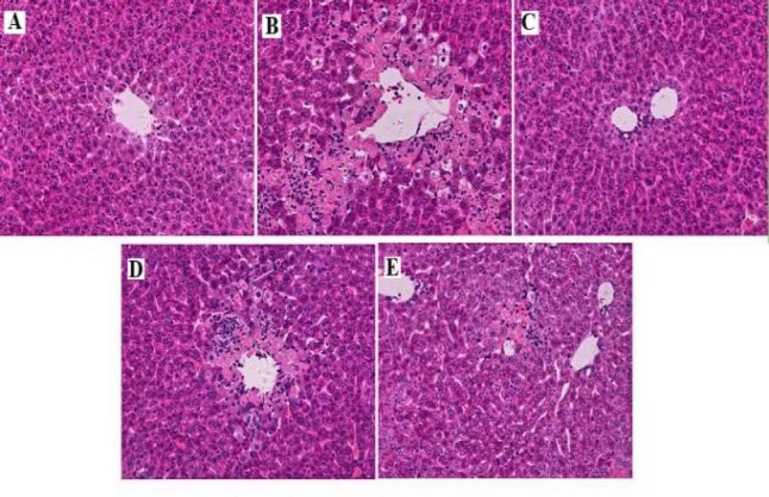
Effect of HAAD on the histopathological morphology of rats liver as assessed by hematoxylin and eosin (H & E) staining (magnification X400). (A) Normal control, (B) CCl_4_ group, (C) Silymarin 100 mg/kg b.w. + CCl_4_, (D) HEAD 100 mg/kg b.w. + CCl_4_, and (E) HAAD 200 mg/kg b.w. + CCl_4_

Histopathological examination of normal group (Figure 9A) animals showed normal layout with distinct cells, visible central vein and sinusoidal spaces. In contrast, the CCl_4_ group showed liver injuries such as moderate to severe necrosis around the central vein, neutrophils infiltration, ballooning degeneration and loss of cellular bourgeons (Figure 9B). Rats treated with silymarin showed almost normal architecture with uniform sinusoids (Figure 4C). Treatment with HAAD (100 mg/kg (Figure. 9D) and 200 mg/kg (Figure 9E)) revealed a relatively normal pattern with a mild degree of necrosis and inflammatory cells infiltration compared to CCl_4_ group. 

## Discussion

Previous studies on *A. dracunculus* indicate that this plant has antibacterial, antiplatelet, anti-inflammatory, antihyperglycemic, gastroprotective and antioxidant activities (Aglarova, 2006 ▶, Benli et al., 2007 ▶, Kheterpal et al., 2010 ▶, Lopes-Lutz et al., 2008 ▶, Shahriyary and Yazdanparast, 2007 ▶). 

In the present experiment, the antioxidant activity of HAAD and the possible mechanisms were investigated by investigation of HEAD reducing power, as well as its DPPH and ABTS radicals scavenging activity. The hepatoprotective effect was also estimated using a model of hepatotoxicity induced by CCl_4_ in rats. In addition, an attempt was made to realize the correlation between antioxidant and hepatoprotective activity.

FRAP is one mechanism for operation of antioxidants and may serve as a significant marker of potential antioxidant activity of antioxidants (Canabady-Rochelle et al., 2015 ▶). In this study, the extract showed a good concentration-dependent reducing power, which was consistent with the findings of Rajabian et al. (Rajabian et al., 2016 ▶).

DPPH and ABTS radical scavenging methods are general spectrophotometric procedures for assessment of antioxidant capacities of chemicals (Schaich et al., 2015 ▶). In this assay, HAAD showed pronounced DPPH radical and ABTS radical scavenging activity, and it was almost as effective as ascorbic acid at similar concentrations. These results are similar to those of Bahramkia et al. (Bahramkia et al., 2008)

CCl_4_ is a potent hepatotoxin known for inducing hepatotoxicity features in animals that are similar to those of acute hepatitis in humans (Li et al., 2015 ▶). CCl_4_ is metabolized by cytochrome P-450 and converted to trichloromethyl and trichloromethyl peroxy radicals which initiate peroxidation of polyunsaturated fatty acid constituents of various membranes with secondary damage, severe enzymatic dispensations, and increased MDA production (Zhou et al., 2010 ▶). Elevation in serum ALT, AST and ALP activity induced by CCl_4_ have been ascribed to hepatic structural damage because these enzymes are generally localized in the cytosol and released into the blood circulation after cellular damage (Huang et al., 2012 ▶. Shah et al., 2015 ▶). In the present study, the levels of all these enzymes increased in CCl_4_ group (CCl_4_-treated group) indicating liver damage induced by CCl_4_. Bilirubin, a product of heme degradation, is a most helpful clinical indicator of necrosis severity (Vuda et al., 2012 ▶). As shown in results, total bilirubin level significantly elevated in CCl_4_ group exhibiting severe injury induced by CCl_4_. Therefore, pretreatment of rats with 50, 100 and 200 mg/kg of HAAD decreased elevated levels of ALT, AST, ALP and TB suggesting that the plant extract effectively protected the animals against CCl_4_-induced hepatotoxicity. Restoration of serum enzyme activities to normal amounts in rats after treatment with HAAD shows prohibition of the leakage of intracellular enzymes by maintaining the integrity of liver cells membrane. In this experiment, CCl_4_ also decreased serum TP while HAAD 50, 100 and 200 mg/kg of HAAD significantly restored the protein synthesis. The hepatoprotective effect of HAAD was further investigated by the histopathological examinations. HAAD at different doses offered hepatoprotection, but 200 mg/kg of HEAD was more effective than the other doses.

Lipid peroxidation is a major index of oxidative stress. Elevated levels of liver MDA induced by CCl_4_ imply enhanced lipid peroxidation which leads to hepatocellular damage and failure of natural antioxidant defense system to prevent overproduction of free radicals (Pareek et al., 2013 ▶). One of the important antioxidant mechanisms is free radical scavenging activity that interferes with the chain reaction of lipid peroxidation (Kepekçi et al., 2013 ▶). In this study, treatment with 100 and 200 mg/kg of HEAD reduced lipid peroxidation by diminishing MDA levels, exhibiting the free radical scavenging activity of tarragon under *in vivo* conditions. This claim was supported by the results of the free radical scavenging activity of HAAD under *in vitro* conditions using DPPH and ABTS tests as mentioned above.

It has been presumed that one of the main underlying mechanisms of CCl_4_-induced liver damage is formation of lipid peroxides by free radicals produced by CCl_4_. Hence, the antioxidant activity or the prohibition of the production of free radicals is important in the defense against CCl_4_-induced hepatotoxicity (Zeashan et al., 2008 ▶). The body has an impressive defense system to hinder and nullify the free radicals-induced harm. This system possesses a collection of endogenous antioxidant enzymes like SOD and CAT. These enzymes establish a supportive group of defense system against ROS (Amresh et al., 2007 ▶). In hepatotoxicity induced by CCl_4_, the balance between ROS formation and these antioxidant defenses may be vanished and oxidative stress occurs; oxidative stress during a series of events, disturbs the cellular functions leading to hepatic damage. Diminished activities of SOD, CAT and GST indicate the hepatic injury in the rats administered with CCl_4_ but treatment with 50, 100 and 200 mg/kg of HAAD showed significant elevation in the level of these enzymes, which reflects the antioxidant activity of *A. dracunculus* extract. Concerning non-enzymatic antioxidants, GSH is a crucial determinant of tissue proneness to oxidative damage and the depletion of hepatic GSH has been shown to be related with an enhanced toxicity to chemicals, including CCl_4_ (Jiang et al., 2015 ▶). In this study, a decrease in liver tissue GSH level was observed in CCl_4_-treated groups. The increase in hepatic GSH level in the animals treated with 50, 100 and 200 mg/kg of HAAD may be due to de novo GSH synthesis or GSH regeneration.

The *in vitro* and *in vivo* antioxidant activities of HAAD may be due to the presence of phenolic compounds and flavonoids in the extract for which antioxidant and hepatoprotective activities have been recognized (Fuda et al., 2015 ▶). However, further studies on the active compounds and their biochemical mechanisms which may attribute to the antioxidant and hepatoprotective effects of *Artemisia dracunculus* L., are necessary to be done.

In the present study, hydro-alcoholic extract of *A. dracunculus* restored the increased serum enzyme levels, diminished liver antioxidant markers, and exerted effective antioxidant activity under *in vitro* conditions suggesting that it has hepatoprotective and antioxidant capacities in CCl_4_-intoxicated rats. The hepatoprotective effect of HAAD may be due to decreased lipid peroxidation and ameliorated defense of the hepatocytes against ROS. The histopathological studies also confirmed the activity of the plant extract. However, the protective, curative and antioxidant characteristics of *A. dracunculus* need to be verified using larger numbers of animals, and determining the active ingredient(s) of this plant as well as recognizing their mechanism(s) of action.
